# Supplementary figures and images for: Identification of compounds that cause axonal dieback without cytotoxicity in dorsal root ganglia explants and intervertebral disc cells with potential to treat pain via denervation
Source: PLoS One. 2024 May 2;19(5):e0300254. doi: 10.1371/journal.pone.0300254 (PMC11065314; doi:10.1371/journal.pone.0300254)

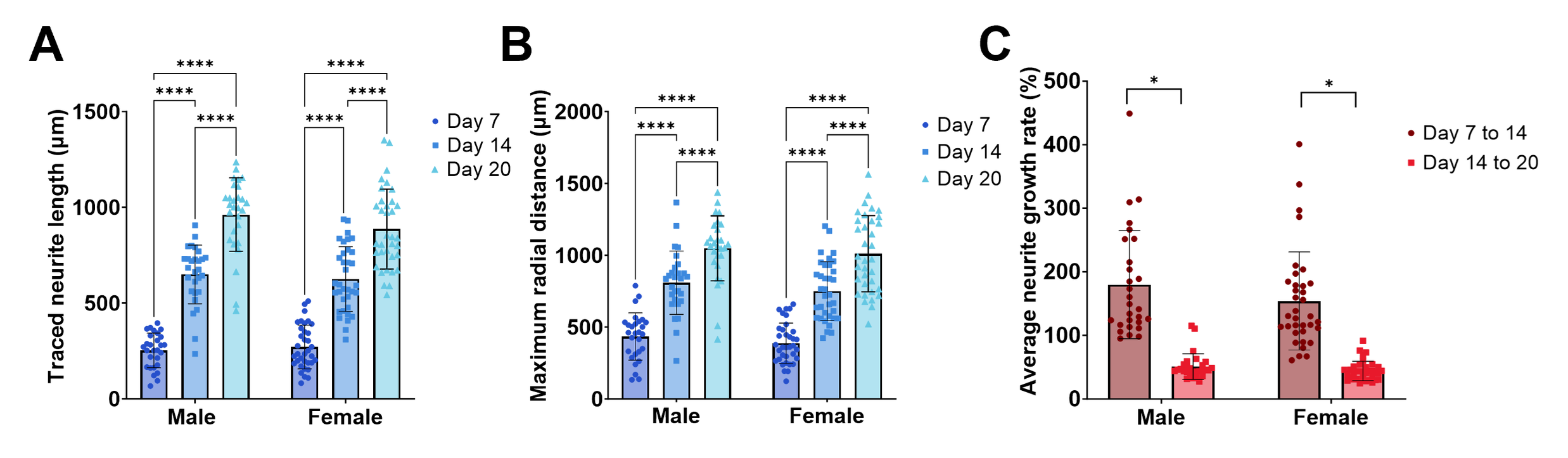

Supplement: S1 Fig — (A) Traced axon lengths and of male rat DRGs and female rat DRGs measured on days 7, 14 and 20 increased significantly over time. No significant differences were detected in traced axon length between male and female DRGs within each day. (B) Similarly, maximum radial distance of male rat DRGs and female rat DRGs increased significantly from days 7, 14 to 20, but no sex differences were detected. (C) Average axon growth rate calculated as the percent difference of traced axon length on day 14 to day 7 and on day 20 to day 14, respectively showed both male and female rat DRGs have significantly higher growth rate between day 7 to 14 compared to growth rate from day 14 to 20. Sex differences in DRG axon growth rate was not detected. (TIF) [file pone.0300254.s003.tif]

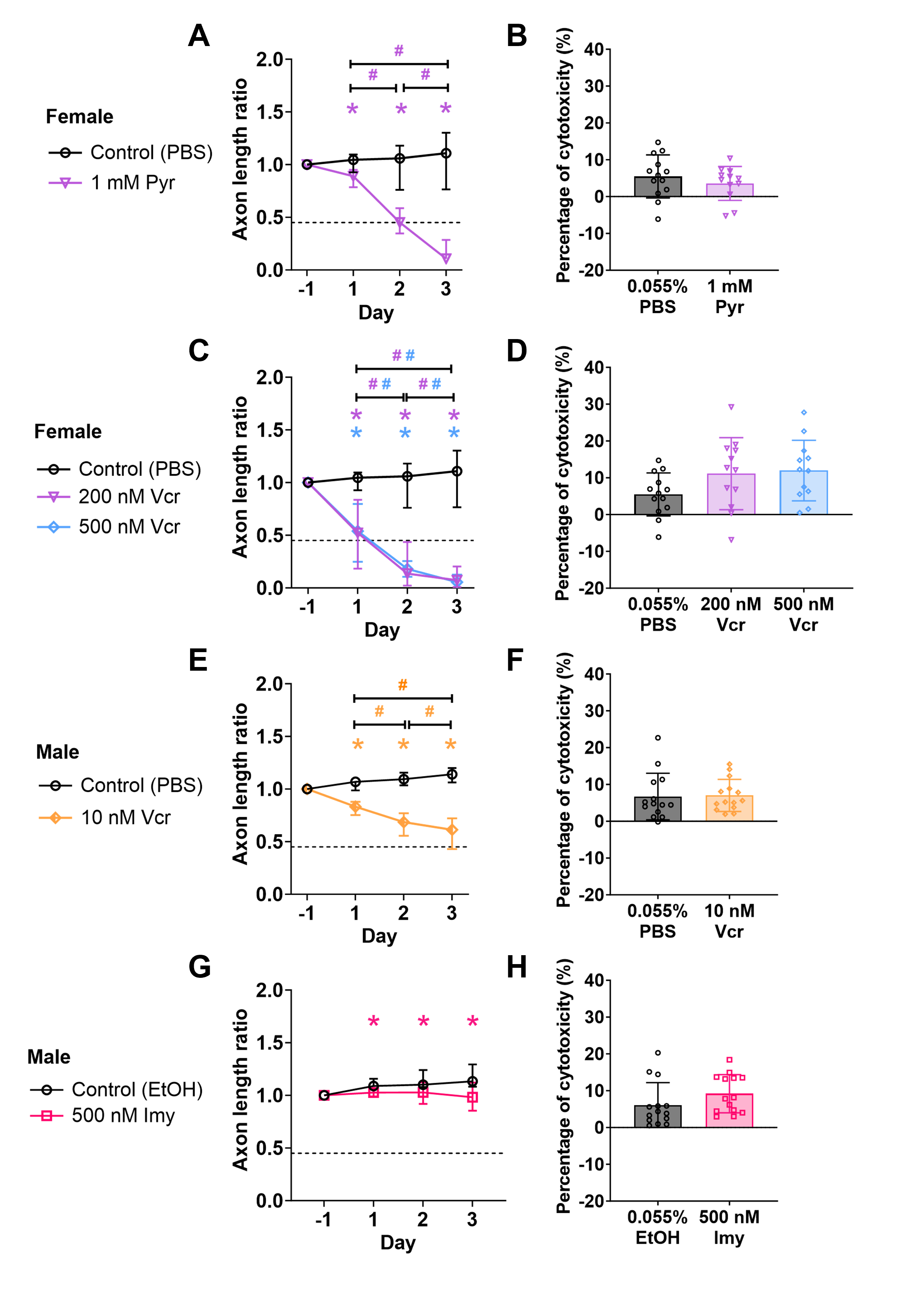

Supplement: S2 Fig — At higher doses, axon length ratio significantly decreased with (A) 1 mM Pyr, (C) 200 nM Vcr and 500 nM Vcr compared to matched solvent controls in female rat DRGs as expected. Testing lower doses, (E) 10 nM Vcr and (G) 500 nM Imy also had significantly lowered axon length ratio compared to matched controls. However, 500 nM Imy did induce significant change in axon length ratio over time which suggests 500 nM Imy prevents axonal outgrowth without dieback. (B, D, F, H) LDH assay did not detect any significance in percentage of cytotoxicity levels of 1 mM Pyr, 200 nM Vcr, 500 nM Vcr and 500 nM Imy. The asterisk (*) symbol represents significant difference in axon length ratio compared to control on each day while the hashtag (#) symbol represents significant difference in axon length ratio between days. Axon length ratio data shows the median and interquartile range of DRG replicates tested per group. Cytotoxicity data shows a scatter plot of each DRG replicate with bar graph centered to the mean and error bars representing standard deviation. EtOH: ethanol, Imy: Ionomycin, Pyr: pyridoxine, Vcr: vincristine sulfate. (TIF) [file pone.0300254.s004.tif]

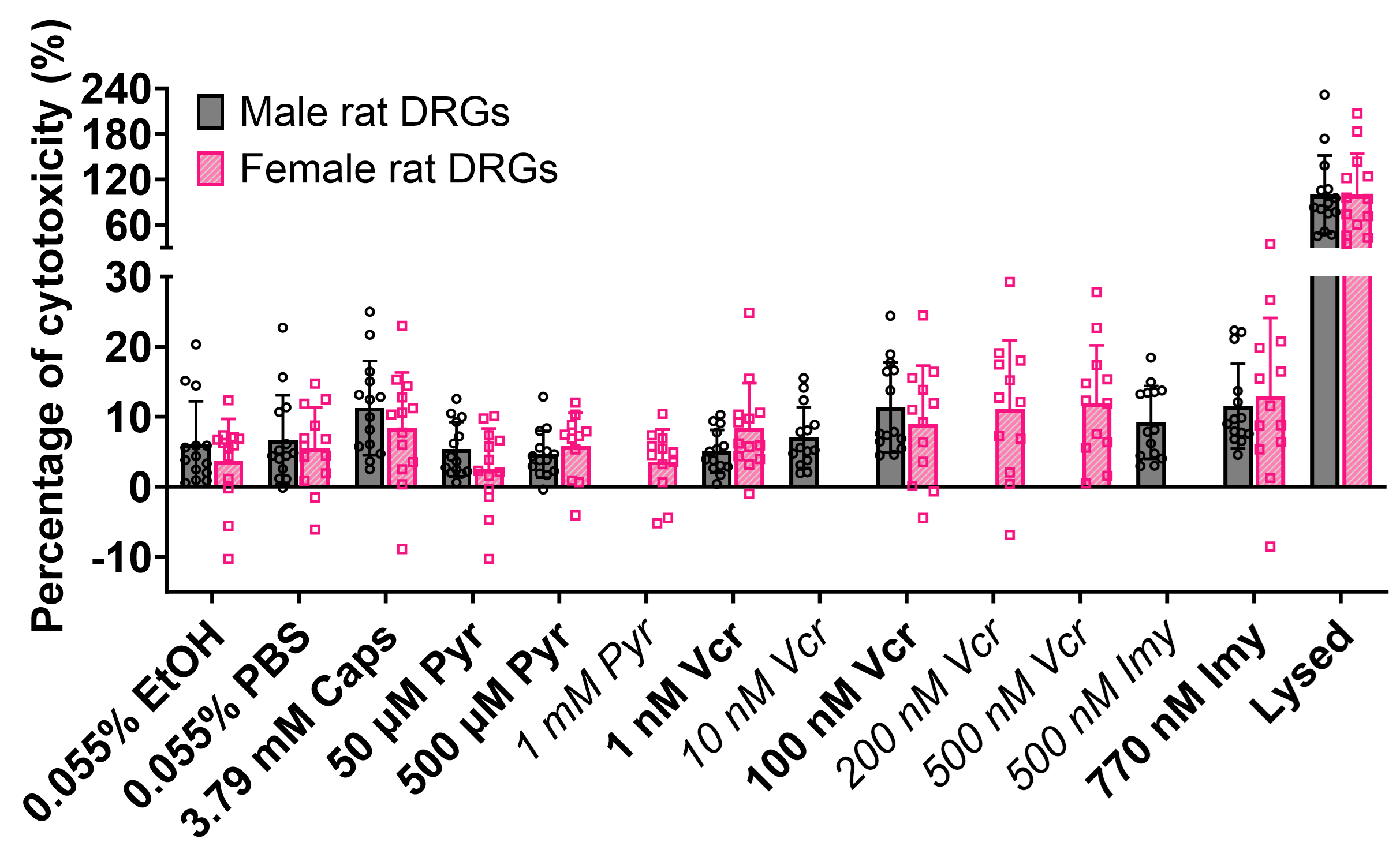

Supplement: S3 Fig — All DRGs treated with solvent control and compounds of interest have significantly lower percentage of cytotoxicity compared to lysed DRGs across all concentrations. The scatter plot shows value from DRG replicate and mean and standard deviation. (TIF) [file pone.0300254.s005.tif]

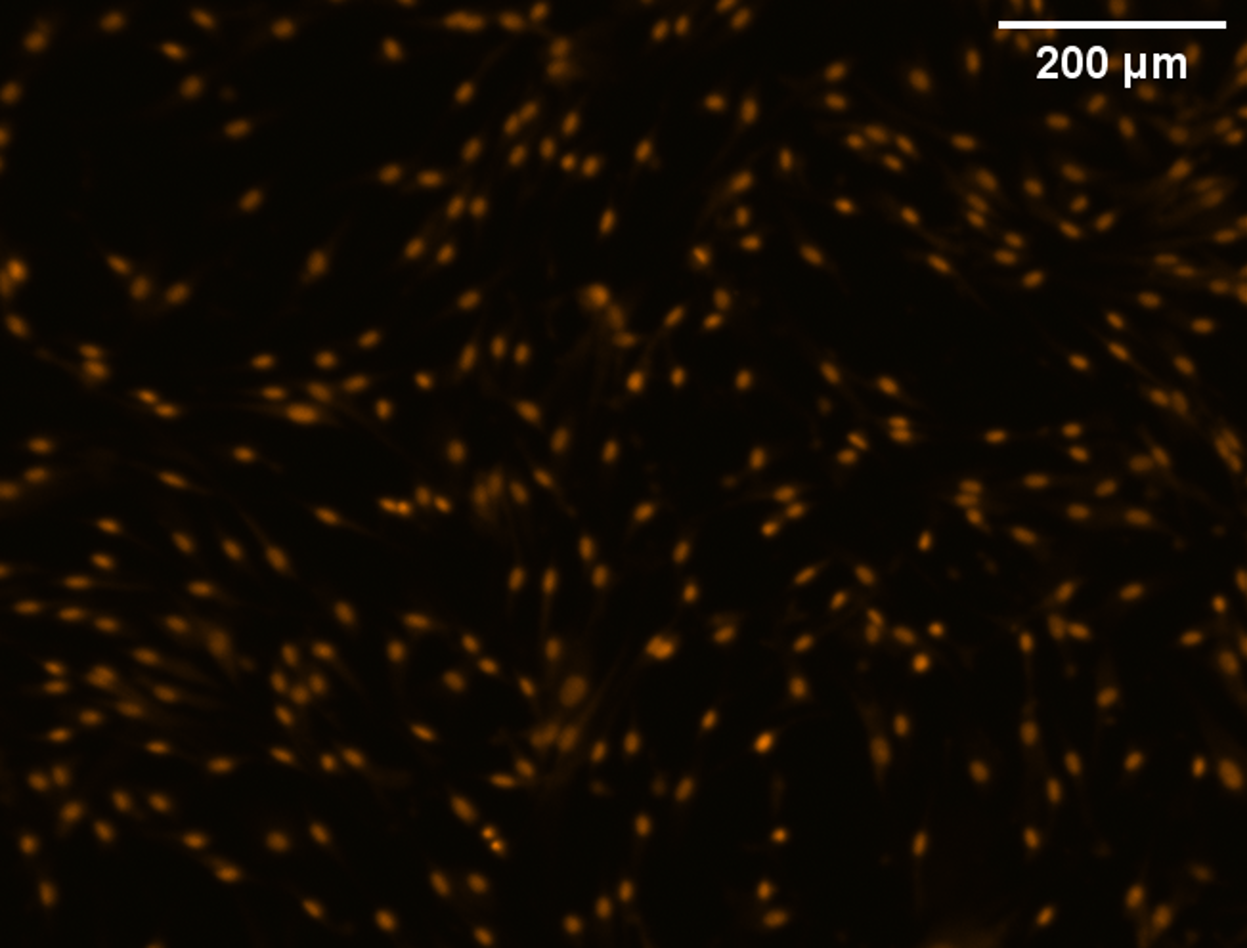

Supplement: S4 Fig — Human NP cells were incubated with media for three days. Then, cells were lysed with 70% Ethanol for 10 minutes at 37°C, stained with LIVE/DEAD staining solution for 30 minutes at room temperature then rinsed with 1X PBS and imaged using Cytation plate imager (Agilent). This well was used as a positive control for dead cells and to determine the image settings for the RFP channel. (TIF) [file pone.0300254.s006.tif]

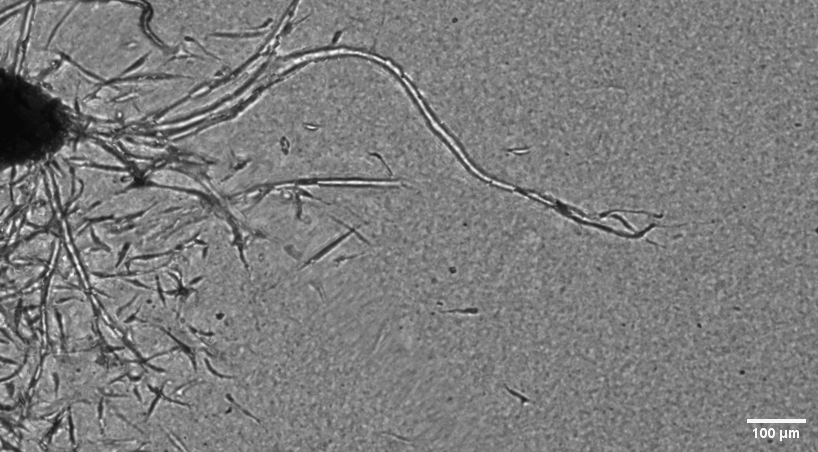

Supplement: S1 Video — Series of brightfield images before and after on days 1, 2 and 3 post-treatment show axonal growth and normal morphology with smooth, continuous axonal paths in EtOH vehicle control group. (GIF) [file pone.0300254.s007.gif]

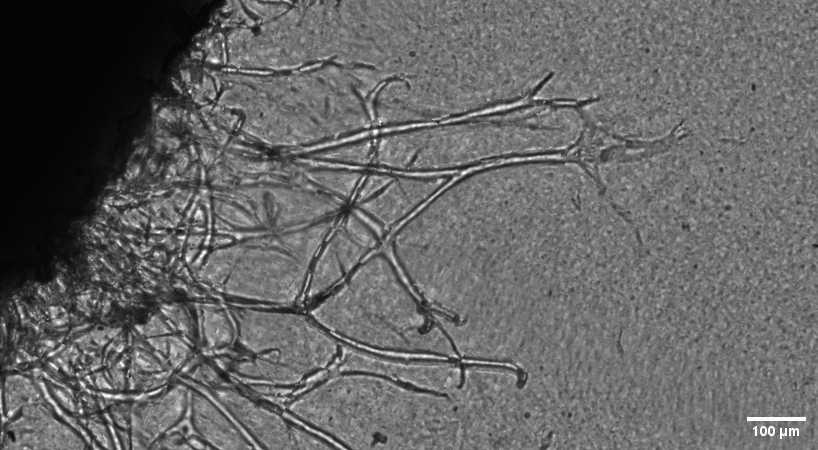

Supplement: S2 Video — Series of brightfield images before and after on days 1, 2 and 3 post-treatment show axonal growth and normal morphology with smooth, continuous axonal paths in PBS vehicle control group. (GIF) [file pone.0300254.s008.gif]

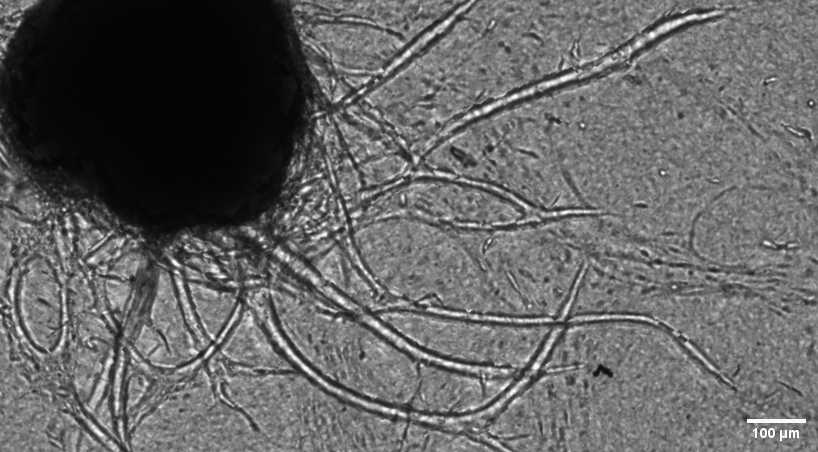

Supplement: S3 Video — Series of brightfield images before and after on days 1, 2 and 3 post-treatment with 3.79 mM Caps show progression of axonal dieback towards the soma with rounded nerve endings. (GIF) [file pone.0300254.s009.gif]

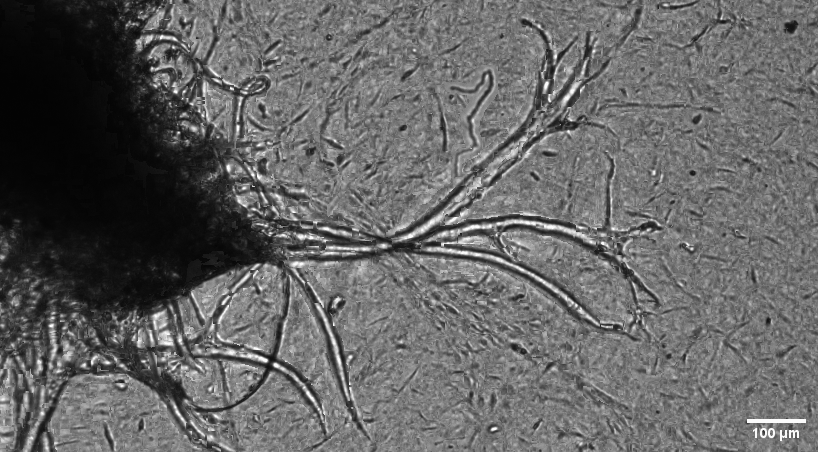

Supplement: S4 Video — Series of brightfield images before and after on days 1, 2 and 3 post-treatment show normal axonal morphology and growth when treated with 50 μM Pyr. (GIF) [file pone.0300254.s010.gif]

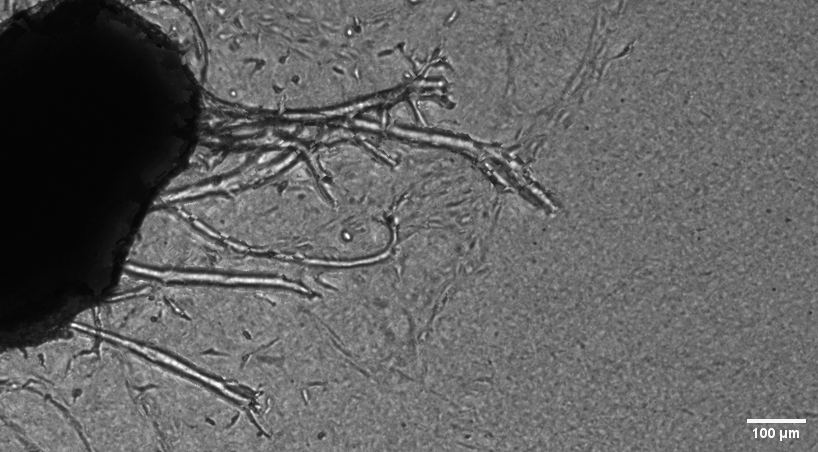

Supplement: S5 Video — Series of brightfield images before and after on days 1, 2 and 3 post-treatment show progression of axonal dieback towards the soma with rounded nerve endings when treated with 500 μM Pyr. Axons appear thicker and have bulbed endings. (GIF) [file pone.0300254.s011.gif]

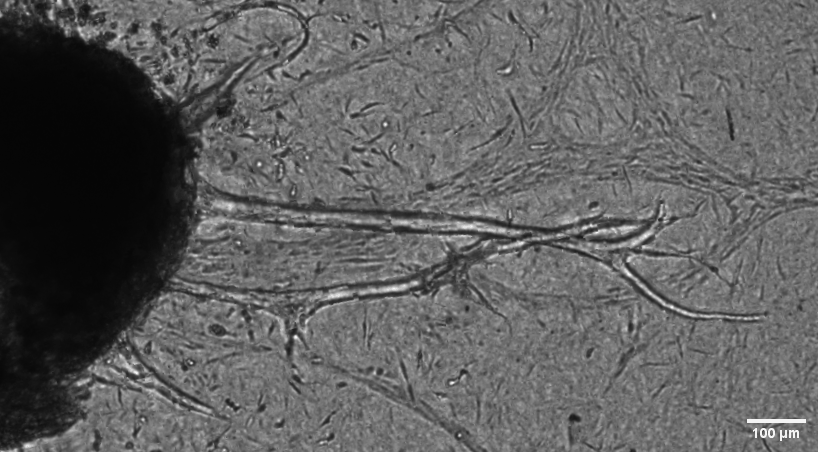

Supplement: S6 Video — Series of brightfield images before and after on days 1, 2 and 3 post-treatment show progression of axonal dieback towards the soma with rounded nerve endings when treated with 1 mM Pyr. Axons completely disappear on day 3 post-treatment. (GIF) [file pone.0300254.s012.gif]

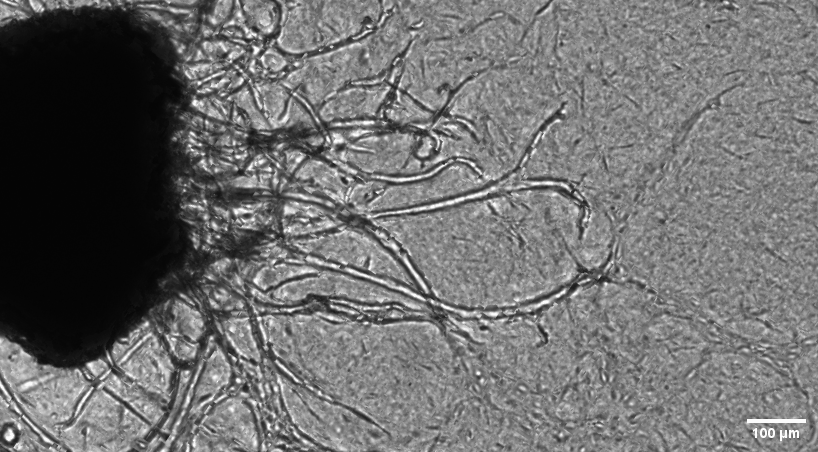

Supplement: S7 Video — Series of brightfield images before and after on days 1, 2 and 3 post-treatment show progression of axonal beading but no reduction in axonal length or presence of fragmentation when when treated with 1 nM Vcr. (GIF) [file pone.0300254.s013.gif]

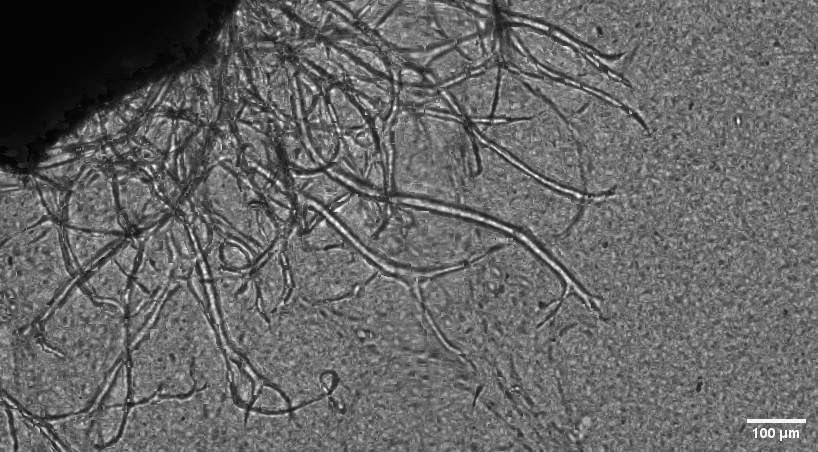

Supplement: S8 Video — Series of brightfield images before and after on days 1, 2 and 3 post-treatment show progression of axonal dieback towards the soma with axonal fragmentation when treated with 100 nM Vcr. (GIF) [file pone.0300254.s014.gif]

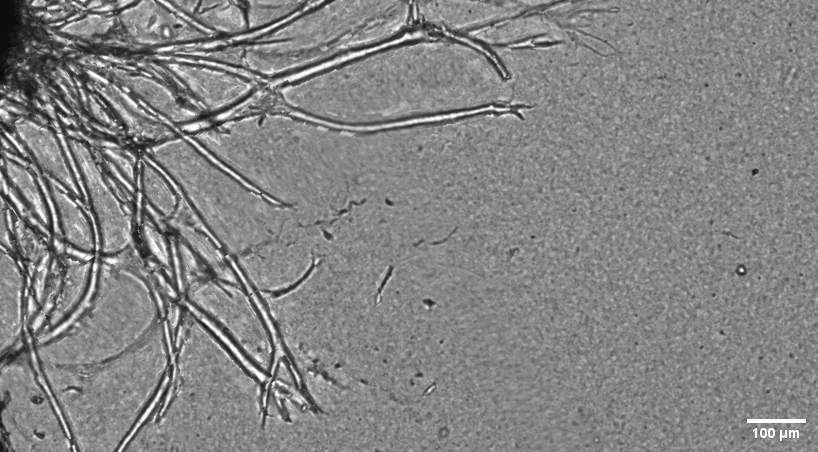

Supplement: S9 Video — Series of brightfield images before and after on days 1, 2 and 3 post-treatment show progression of axonal dieback towards the soma with axonal fragmentation when treated with 200 nM Vcr. (GIF) [file pone.0300254.s015.gif]

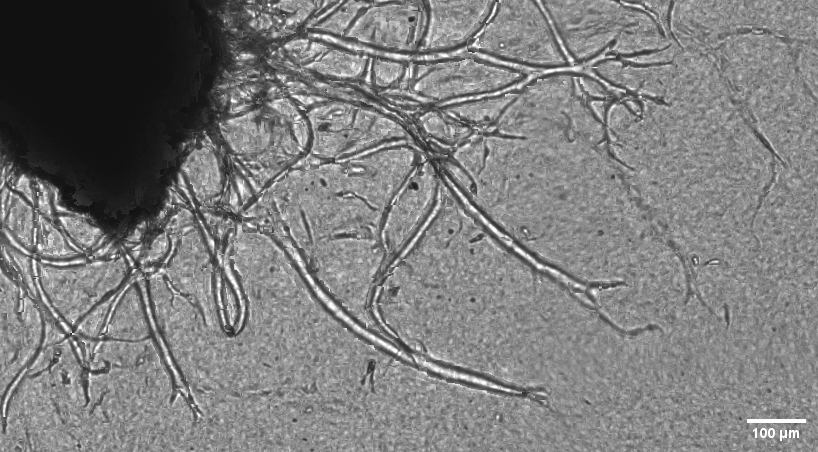

Supplement: S10 Video — Series of brightfield images before and after on days 1, 2 and 3 post-treatment show progression of axonal dieback towards the soma with axonal fragmentation when treated with 500 nM Vcr. (GIF) [file pone.0300254.s016.gif]
