# Supplementary material for: Identification of compounds that cause axonal dieback without cytotoxicity in dorsal root ganglia explants and intervertebral disc cells with potential to treat pain via denervation
Source: PLoS One. 2024 May 2;19(5):e0300254. doi: 10.1371/journal.pone.0300254 (PMC11065314; doi:10.1371/journal.pone.0300254)
Supplement: Attachment — Submitted filename: Response to Reviewers.docx [file pone.0300254.s017.docx]

# Journal requirements:

When submitting your revision, we need you to address these additional requirements.

1. Please ensure that your manuscript meets PLOS ONE's style requirements, including those for file naming. The PLOS ONE style templates can be found at

<https://journals.plos.org/plosone/s/file?id=wjVg/PLOSOne_formatting_sample_main_body.pdf> and

<https://journals.plos.org/plosone/s/file?id=ba62/PLOSOne_formatting_sample_title_authors_affiliations.pdf>

**Thank you for reviewing the formatting. We have made the necessary changes formatting changes in the revised manuscript by adjusting the headings, title page and authors list.**

2. Note from Emily Chenette, Editor in Chief of PLOS ONE, and Iain Hrynaszkiewicz, Director of Open Research Solutions at PLOS: Did you know that depositing data in a repository is associated with up to a 25% citation advantage (<https://doi.org/10.1371/journal.pone.0230416>)? If you’ve not already done so, consider depositing your raw data in a repository to ensure your work is read, appreciated and cited by the largest possible audience. You’ll also earn an Accessible Data icon on your published paper if you deposit your data in any participating repository (<https://plos.org/open-science/open-data/#accessible-data>).

**Thank you for the thoughtful recommendation. We have included the relevant datasets in the supplementary information.**

3. Please include your tables as part of your main manuscript and remove the individual files. Please note that supplementary tables (should remain/ be uploaded) as separate "supporting information" files.

**Table 1 has been added to the main manuscript in Lines 441 to 445.**

4. Thank you for stating the following financial disclosure:

“RAW received the NSF CAREER award (NSF CAREER1846857) from the National Science Foundation (NSF) which provided funds to conduct this research. RAW, FSL, UNN received funding by NSF CAREER1846857. EJM received funding from NSF Research Experiences for Undergraduates (REU) program EEC 2050587 as a part of this work.”

Please state what role the funders took in the study.  If the funders had no role, please state: "The funders had no role in study design, data collection and analysis, decision to publish, or preparation of the manuscript."

If this statement is not correct you must amend it as needed.

Please include this amended Role of Funder statement in your cover letter; we will change the online submission form on your behalf.

**The statement above is correct. We have included the statement to request the revision on the online submission form in the cover letter.**

5. Thank you for stating the following in the Acknowledgments Section of your manuscript:

“We would like to thank the National Science Foundation (NSF) for providing funds to conduct this research (NSF CAREER1846857). We would also like to acknowledge the NSF Research Experiences for Undergraduates (REU) program EEC 2050587 for supporting part of this work.”

We note that you have provided funding information that is currently declared in your Funding Statement. However, funding information should not appear in the Acknowledgments section or other areas of your manuscript. We will only publish funding information present in the Funding Statement section of the online submission form.

Please remove any funding-related text from the manuscript and let us know how you would like to update your Funding Statement. Currently, your Funding Statement reads as follows:

“RAW received the NSF CAREER award (NSF CAREER1846857) from the National Science Foundation (NSF) which provided funds to conduct this research. RAW, FSL, UNN received funding by NSF CAREER1846857. EJM received funding from NSF Research Experiences for Undergraduates (REU) program EEC 2050587 as a part of this work.”

Please include your amended statements within your cover letter; we will change the online submission form on your behalf.

**Thank you for stating the suggested edits. We have removed all funding-related text from the manuscript. The Funding Statement section of the online submission form is correct, and the statement "The funders had no role in study design, data collection and analysis, decision to publish, or preparation of the manuscript." should be added for clarification, as suggested in Comment #4 above.**

6. We note that you have indicated that data from this study are available upon request. PLOS only allows data to be available upon request if there are legal or ethical restrictions on sharing data publicly. For more information on unacceptable data access restrictions, please see <http://journals.plos.org/plosone/s/data-availability#loc-unacceptable-data-access-restrictions>.

In your revised cover letter, please address the following prompts:

a) If there are ethical or legal restrictions on sharing a de-identified data set, please explain them in detail (e.g., data contain potentially sensitive information, data are owned by a third-party organization, etc.) and who has imposed them (e.g., an ethics committee). Please also provide contact information for a data access committee, ethics committee, or other institutional body to which data requests may be sent.

b) If there are no restrictions, please upload the minimal anonymized data set necessary to replicate your study findings as either Supporting Information files or to a stable, public repository and provide us with the relevant URLs, DOIs, or accession numbers. For a list of acceptable repositories, please see <http://journals.plos.org/plosone/s/data-availability#loc-recommended-repositories>.

We will update your Data Availability statement on your behalf to reflect the information you provide.

**We support the idea of open access data for scientific progress. Hence, we upload the data set for axon length measurements, DRG cytotoxicity, NP cell viability and NP cell metabolic activity in the form of spreadsheets in the supporting information to .**

7. Please include a separate caption for each figure in your manuscript.

**The figure captions for each figure have been added to the manuscript in the appropriate locations.**

8. We note that Figure 1 in your submission contain copyrighted images. All PLOS content is published under the Creative Commons Attribution License (CC BY 4.0), which means that the manuscript, images, and Supporting Information files will be freely available online, and any third party is permitted to access, download, copy, distribute, and use these materials in any way, even commercially, with proper attribution. For more information, see our copyright guidelines: <http://journals.plos.org/plosone/s/licenses-and-copyright>.

We require you to either (1) present written permission from the copyright holder to publish these figures specifically under the CC BY 4.0 license, or (2) remove the figures from your submission:

1. You may seek permission from the original copyright holder of Figure 1 to publish the content specifically under the CC BY 4.0 license.

We recommend that you contact the original copyright holder with the Content Permission Form (<http://journals.plos.org/plosone/s/file?id=7c09/content-permission-form.pdf>) and the following text:

“I request permission for the open-access journal PLOS ONE to publish XXX under the Creative Commons Attribution License (CCAL) CC BY 4.0 (<http://creativecommons.org/licenses/by/4.0/>). Please be aware that this license allows unrestricted use and distribution, even commercially, by third parties. Please reply and provide explicit written permission to publish XXX under a CC BY license and complete the attached form.”

Please upload the completed Content Permission Form or other proof of granted permissions as an "Other" file with your submission.

In the figure caption of the copyrighted figure, please include the following text: “Reprinted from [ref] under a CC BY license, with permission from [name of publisher], original copyright [original copyright year].”

2. If you are unable to obtain permission from the original copyright holder to publish these figures under the CC BY 4.0 license or if the copyright holder’s requirements are incompatible with the CC BY 4.0 license, please either i) remove the figure or ii) supply a replacement figure that complies with the CC BY 4.0 license. Please check copyright information on all replacement with figures and update the figure caption with source information. If applicable, please specify in the figure caption text when a figure is similar but not identical to the original image and is therefore for illustrative purposes only.

**BioRender was unable to provide a CC BY 4.0 license; therefore, we have chosen to remove Figure 1 below from the publication.**

9. Please include captions for your Supporting Information files at the end of your manuscript, and update any in-text citations to match accordingly. Please see our Supporting Information guidelines for more information: <http://journals.plos.org/plosone/s/supporting-information>.

**We have added the captions for the supporting Information at the end of the manuscript and updated the in-text citations.**

10. We notice that your supplementary figures are uploaded with the file type 'Figure'. Please amend the file type to 'Supporting Information'. Please ensure that each Supporting Information file has a legend listed in the manuscript after the references list.

**We will do this upon upload.**

# Reviewer comments

Reviewer's Responses to Questions

**Comments to the Author**

1. Is the manuscript technically sound, and do the data support the conclusions?

The manuscript must describe a technically sound piece of scientific research with data that supports the conclusions. Experiments must have been conducted rigorously, with appropriate controls, replication, and sample sizes. The conclusions must be drawn appropriately based on the data presented.

Reviewer #1: Yes

Reviewer #2: Yes

2. Has the statistical analysis been performed appropriately and rigorously?

Reviewer #1: Yes

Reviewer #2: Yes

3. Have the authors made all data underlying the findings in their manuscript fully available?

The [PLOS Data policy](https://urldefense.com/v3/__http:/www.plosone.org/static/policies.action*sharing__;Iw!!PvXuogZ4sRB2p-tU!HAjZgAh6xWm6hVPunDKK9m7igBbLlsWFr99QzU-WSKlNhIuuIXB6cryZ5Px6cFEvKlnkzDTAvYX9VXXvVbFd$) requires authors to make all data underlying the findings described in their manuscript fully available without restriction, with rare exception (please refer to the Data Availability Statement in the manuscript PDF file). The data should be provided as part of the manuscript or its supporting information, or deposited to a public repository. For example, in addition to summary statistics, the data points behind means, medians and variance measures should be available. If there are restrictions on publicly sharing data—e.g. participant privacy or use of data from a third party—those must be specified.

Reviewer #1: Yes

Reviewer #2: Yes

4. Is the manuscript presented in an intelligible fashion and written in standard English?

PLOS ONE does not copyedit accepted manuscripts, so the language in submitted articles must be clear, correct, and unambiguous. Any typographical or grammatical errors should be corrected at revision, so please note any specific errors here.

Reviewer #1: Yes

Reviewer #2: Yes

5. Review Comments to the Author

Please use the space provided to explain your answers to the questions above. You may also include additional comments for the author, including concerns about dual publication, research ethics, or publication ethics. (Please upload your review as an attachment if it exceeds 20,000 characters)

Reviewer #1: The work describes the effects of pyridoxine (Pyr), vincristine sulfate (Vcr) and ionomycin (Imy) on DRG explant axonal dieback and cytotoxicity. The experiments and results have been described very clearly.
I have only two comments:

-As the Authors stated, the used DRG explant model has the limitation that both the neuronal cell bodies and the axons have been exposed to the tested compounds. I wonder whether this model is physiologically relevant to study axonal dieback. While this first screening was important, the Authors should include an experiment where the compounds are tested on the axonal compartment only, to make their findings more physiologically relevant.

**Thank you for your comment. There are two main parts to physiological relevance, one is having an intact DRG explant with support cells, the second is having isolation of the soma and neurites. While there are some systems that isolate the DRG cell body and neurites, they are 2D in nature and involve dissociated DRGs, thereby eliminating support cells. Thus, they only address one aspect of physiological relevance. We believe maintaining an intact DRG is more important than soma and neurite isolation as we maintain support cells that will likely impact function. Despite the identified limitation, our unique approach strikes a balance between addressing experimental challenges and maintaining a realistic representation of the physiological context, contributing to the robustness and applicability of our findings.**

-All the analysis have been done on bright-field images. It would be meaningful complementing with fluorescence analysis, using axonal markers, to have a better understanding of the axon morphology (axon blebbing, axon retraction).

**Thank you for the suggestion. While we recognize the valuable insight that fluorescence analysis would provide, we believe this would be redundant since the brightfield images in Figure 1 (previously Figure 2) already offer a clear visualization of axonal morphology. Our choice of brightfield imaging as the primary method for axonal analysis stems from its ability to effectively capture changes in axonal morphology and length over time without the use fixation and staining. This aligns with our experimental goals while eliminating the need for duplicative analysis. To more clearly show morphological changes in the axons, we have added a new Figure 2 that zooms into and enlarges the axons in the brightfield images. In addition, we have provided supplementary GIF image sequences that clearly show the progression of axonal retraction or axonal blebbing in DRG axons after treatment.**

Reviewer #2: The manuscript investigates potential application of pyridoxine (Pyr) and vincristine sulfate (Vcr) to cause local denervation and alleviate pain induced by aberrant nerve growth in tissue. The author utilized rat DRG explant hydrogel culture platform for axonal dieback compound screening under four drugs: capsaicin (Caps), Pyr, Vcr, and ionomycin (Imy). Axonal length was measured before and after drug treatment to calculate axon length ratio. To assess cytotoxicity of the drugs, DRG lactate dehydrogenase (LDH) activity was measured post drug treatment. Drug toxicity to human nucleus pulposus (NP) cells was also studied in vitro through cell viability kits. Results showed that Pyr and Vcr was able to induce reduction in axonal length at high concentration while Imy produced slower axonal dieback. Imy, Vcr and Pyr had only minimal neurotoxicity. Further, neither Pyr nor Vcr triggered NP cell death or affected cellular metabolic activity. Overall, the research is exciting and brought up new axon dieback candidates to treat pain without induced cytotoxicity to intervertebral disc cells, but there are some concerns need to be addressed:

1. 3.79 mM Caps was used as positive control for the study, but there lacks detailed description how the concentration was calculated. Although 8% Caps is used in commercial patch, it’s better to investigate the effects of different concentration on DRG explant to strengthen the advantages of Pyr and Vcr over Caps.

**Thank you for seeking clarification. We specified in Lines 127 to 129: “**Based on the concentration and surface area used in 8% topical Caps dermal patch [1], the concentration used was scaled down to the surface area of the hydrogel platform, and thus was diluted to 3.79 mM Caps as a positive control for axonal degeneration.”. **Since capsaicin is a widely known compound that induces axonal degeneration to alleviate pain, the axonal dieback properties of capsaicin are well-documented [2, 3]. The 8% w/w capsaicin concentration was selected to be scaled to match the area of the hydrogels in our culture platform because it is the highest concentration currently on the market to treat pain via denervation [2].**

**The objective of this study is to leverage capsaicin as a widely used axonal dieback compound as a positive axonal dieback control for identifying novel compounds to repurpose as axonal dieback compounds. Thus, the rate and degree of axonal length reduction of compounds that meet or exceed capsaicin performance in our platform may have the potential efficacy in pain alleviation via denervation. To clearly indicate whether the test compounds meet the capsaicin threshold, we have added a straight line (minimum threshold for axon length ratio at 0.45 determined by capsaicin retraction) across the axon length ratio graphs in Figure 3. We have added a clarifying statement in Lines 327-328:** “To identify novel axonal dieback compounds that may be able to alleviate pain like capsaicin, an axon length ratio of 0.45 was used as a minimum criterion for selection.”

2. The image quality of DRG outgrowth (Figure 2) is low and it’s hard to visualize the axons.

**Thank you for your comment. To resolve this issue, we have added a new Figure 2 (above) that enlarges the region of interest to clearly visualize axon morphology of control healthy axons and degenerated axons after Pyr and Vcr treatment. In addition to this, we have uploaded a GIF of the image sequence that clearly shows the progression of axonal changes after treatment.**

3. Could the author specify how many axons were included for each DRG explant piece for axonal length (ratio) measurement? Meanwhile, it’s unclear if the axonal length referred to the maximum axonal length or to the average length of several selected axons. If only several axons were selected, could the author clarify the criteria of selection? How did the author make sure the exact same axon was captured before and after drug treatment to calculate the axonal length change (ratio)?

**Thank you for seeking clarification regarding the axon length analysis.**

**Our methodology involved compiling the series of DRG brightfield images collected pre-treatment and on days 1, 2, and 3 post-treatments into a montage using ImageJ. Subsequently, we employed SimpleNeuriteTracer to trace three to 12 randomly selected individual axon paths in each DRG. To ensure consistency in the axons traced pre-treatment and post-treatment for calculating the axon length ratio, we adopted a systematic approach. The axons were traced sequentially in the order of the pre-treatment image, followed by the day 1, day 2, and day 3 images. The location of the axon paths was cross-verified based on the axon traced in the pre-treatment image, ensuring the accurate tracing of the same axon throughout the analysis. It is also important to note that our selection process for axon paths was random. We did not impose specific criteria for axon path selection, contributing to the robustness and unbiased nature of our approach.**

**We have added clarifying statements in the Methods section for DRG axon length analysis (lines 199 to 211), as shown below.**

Changes in axon length were used to characterize axonal dieback. Four to 12 continuous axon paths were randomly selected per DRG and manually traced at each timepoint after compounds of interest were added using SimpleNeuriteTracer (SNT) plugin in ImageJ (FIJI) [4]. To ensure the same DRG axons were traced before and after treatment, the brightfield DRG images collected pre-treatment and on Day 1, Day 2 and Day 3 post-treatment were compiled into a montage and the same axons were traced in the image sequence. To identify axonal dieback, the length of each axon path traced on days 1, 2 and 3 post treatment were normalized to axon length pre-treatment (day -1) and reported as “axon length ratio” as shown in Equation 1. The axon length ratio represents the fold change in axon length over time. The average axon length ratio from each DRG was calculated and values above 1.0 indicate axon growth and values below 1.0 indicate axonal dieback. Maximum radial distance was measured using the “straight line tool” and “measure tool” in Image J (FIJI) [5] by drawing a straight perpendicular from the longest axon to the nearest point perpendicular to the soma boundary.

$Axon length ratio= \frac{Axon length ratio post-treat}{Axon length ratio pre-treat}$	(1)

4. The author may consider doing immunostaining of the neurites for higher resolution images and more precise quantification. For example, TUJ staining will allow the visualization of neurite outgrowth and Sholl analysis in ImageJ for average axonal length measurement.

**Thank you for the suggestion. While we recognize the valuable insight TUJ staining could provide, we believe this analysis would be redundant since the brightfield images in Figure 1 offer a clear visualization of axonal morphology and growth. Further, we have added Figure 2 (above) to demonstrate changes more clearly in axonal morphology after Pyr and Vcr treatment. Finally, given the nature of immunohistochemistry, it would require four times as many DRGs to attain temporal data without substantial increase in data.**

5. Instead of having two separate figures, the reviewer would recommend showing the dose-dependent drug effect on axonal length and cytotoxicity in one figure, i.e., combining the Figure 2 and supplementary Figure 2 for clearer interpretation.

**We value the recommendation to combine the figures on axonal length and cytotoxicity but we believe they are better kept separate. Figure 3 is intended to examine sex and does dependent differences side by side. However, higher doses, not tested in both sexes are included in S2 Figure.**

6. Could the author explain why they normalized the LDH activity of DRG explant to lysed DRGs? In addition, the term “neurotoxicity” has to be used carefully since DRG consists of various types of cells.

**Thank you for the suggestion. We agree that the use of term “neurotoxicity” is not suitable since the DRGs consist of various types of cells. We have replaced the term “DRG neurotoxicity” to “DRG cytotoxicity” throughout the manuscript.**

7. The AlamarBlue reduction per cell plot in Figure 4 is a bit confusing. Could the author explain why the replicates in PBS control group had the same value? Some groups seem to have significant higher values compared to control (for example 1mM Pyr vs PBS in Donor1) while not highlighted in the plot. In addition to the 2-way ANOVA test, multiple comparison against control is something the author should consider performing here.

**Thank you for seeking clarification. The AlamarBlue reduction per cell plot illustrates the cell metabolic activity of each compound normalized to the average PBS control. Consequently, the values for the PBS control were normalized to its own value, resulting in identical average values of 1.0. For clarity, we have removed the PBS group bar from the plot.**

**We wanted to discern potential differences between various concentrations within each donor in addition to the PBS control, thus we conducted a 2-way ANOVA with a multiple comparison test comparing all groups within each donor. Notably, the 1 mM Pyr group did not exhibit significantly higher values when compared to the PBS control.**

6. PLOS authors have the option to publish the peer review history of their article ([what does this mean?](https://urldefense.com/v3/__https:/journals.plos.org/plosone/s/editorial-and-peer-review-process*loc-peer-review-history__;Iw!!PvXuogZ4sRB2p-tU!HAjZgAh6xWm6hVPunDKK9m7igBbLlsWFr99QzU-WSKlNhIuuIXB6cryZ5Px6cFEvKlnkzDTAvYX9VY5UvBKc$)). If published, this will include your full peer review and any attached files.


If you choose “no”, your identity will remain anonymous but your review may still be made public.


**Do you want your identity to be public for this peer review?** For information about this choice, including consent withdrawal, please see our [Privacy Policy](https://urldefense.com/v3/__https:/www.plos.org/privacy-policy__;!!PvXuogZ4sRB2p-tU!HAjZgAh6xWm6hVPunDKK9m7igBbLlsWFr99QzU-WSKlNhIuuIXB6cryZ5Px6cFEvKlnkzDTAvYX9VatuxpLo$).

Reviewer #1: No

Reviewer #2: **Yes:** Mikhail Berezin

**References:**

[1] M. Wallace, M. Pappagallo, Qutenza®: a capsaicin 8% patch for the management of postherpetic neuralgia, Expert review of neurotherapeutics 11(1) (2011) 15-27.

[2] P. Anand, K. Bley, Topical capsaicin for pain management: therapeutic potential and mechanisms of action of the new high-concentration capsaicin 8% patch, British journal of anaesthesia 107(4) (2011) 490-502.

[3] G. Jancso, E. Király, F. Joo, G. Such, A. Nagy, Selective degeneration by capsaicin of a subpopulation of primary sensory neurons in the adult rat, Neuroscience letters 59(2) (1985) 209-214.

[4] J. Schindelin, I. Arganda-Carreras, E. Frise, V. Kaynig, M. Longair, T. Pietzsch, S. Preibisch, C. Rueden, S. Saalfeld, B. Schmid, Fiji: an open-source platform for biological-image analysis, Nature methods 9(7) (2012) 676-682.

[5] C.A. Schneider, W.S. Rasband, K.W. Eliceiri, NIH Image to ImageJ: 25 years of image analysis, Nature methods 9(7) (2012) 671-675.
